# The Preservation of Cued Recall in the Acute Mentally Fatigued State: A Randomised Crossover Study

**DOI:** 10.1007/s00268-015-3317-9

**Published:** 2015-11-17

**Authors:** Ian Richard Flindall, Daniel Richard Leff, Neysan Pucks, Colin Sugden, Ara Darzi

**Affiliations:** Division of Surgery and Cancer, St Mary’s Hospital, 10th floor, QEQM, Paddington, London, W2 1NY UK

## Abstract

**Purpose:**

The objective of this study is to investigate the impact of acute mental fatigue on the recall of clinical information in the non-sleep-deprived state. Acute mental fatigue in the non-sleep-deprived subject is rarely studied in the medical workforce. Patient handover has been highlighted as an area of high risk especially in fatigued subjects. This study evaluates the deterioration in recall of clinical information over 2 h with cognitively demanding work in non-sleep-deprived subjects.

**Method:**

A randomised crossover study involving twenty medical students assessed free (presentation) and cued (MCQ) recall of clinical case histories at 0 and 2 h under low and high cognitive load using the N-Back task. Acute mental fatigue was assessed through the Visual Analogue Scale, Stanford Scale and NASA-TLX Mental Workload Rating Scale.

**Results:**

Free recall is significantly impaired by increased cognitive load (*p* < 0.05) with subjects demonstrating perceived mental fatigue during the high cognitive load assessment. There was no significant difference in the amount of information retrieved by cued recall under high and low cognitive load conditions (*p* = 1).

**Discussion:**

This study demonstrates the loss of clinical information over a short time period involving a mentally fatiguing, high cognitive load task. Free recall for the handover of clinical information is unreliable. Memory cues maintain recall of clinical information. This study provides evidence towards the requirement for standardisation of a structured patient handover. The use of memory cues (involving recognition memory and cued recall methodology) would be beneficial in a handover checklist to aid recall of clinical information and supports evidence for their adoption into clinical practice.

## Introduction

In 2004, the World Health Organisation identified fatigue as a leading factor in medical error and injury in healthcare [1]. Current fatigue management comprises work hour’s reduction with interspersed breaks. The Accreditation Council for Graduate Medical Education(ACGME) has twice recommended reductions in work time for medical trainees due to concerns regarding fatigue [2]. In the UK, the working hours legislation was principally introduced as part of the European Working Time Directive (EWTD) [3] with work hours expected to average 48 h by August 2009. Despite these restrictions, fatigue has still been highlighted as a concern within the medical profession with the Joint Commission Patient Safety Advisory Group issuing a Sentinel Event Alert in December 2011 [4].

As healthcare worker fatigue is linked t o patient safety, it has been recommended that healthcare organisations examine processes where patients are “handed off” or transitioned from one caregiver to another. This is an area of risk that is compounded by fatigue [5]. However, there are no reports of studies specifically investigating the impact of acute mental fatigue on the recall of clinical information in the non-sleep-deprived state.

Rather, the majority of fatigue literature focuses on cognitive assessment in the sleep deprived. Examples have included word recollection tasks in Emergency department doctors [6], and assessments on vigilance and reaction times in cognitive tests on year 1 qualified doctors [7]. These demonstrate impaired performance with post shift reduced word recall and reaction times that decline with increased sleep loss. Fatigue studies frequently imply sleep deprivation studies that are typically lab-based or performed around shift work. However, acute mental fatigue produced through high mental workload can also cause impairment in working memory [8], but is less recognised or addressed by the medical profession. Hence, one does not necessarily need to be sleep-deprived to be fatigued, and the impact of fatigue on clinical performance in the non-sleep-deprived state remains unknown.

In this study, we hypothesise that memory cues (cued recall and recognition memory) can improve the recall of clinical information in the acute mentally fatigued, non-sleep-deprived state.

## Method

Ethical approval was granted by Cambridgeshire Research Ethics Committee 1 (Ref: 09/H0304/24). Site-specific approval, sponsorship and funding were provided by Imperial College London.

## Study design

**Fig. 1:**
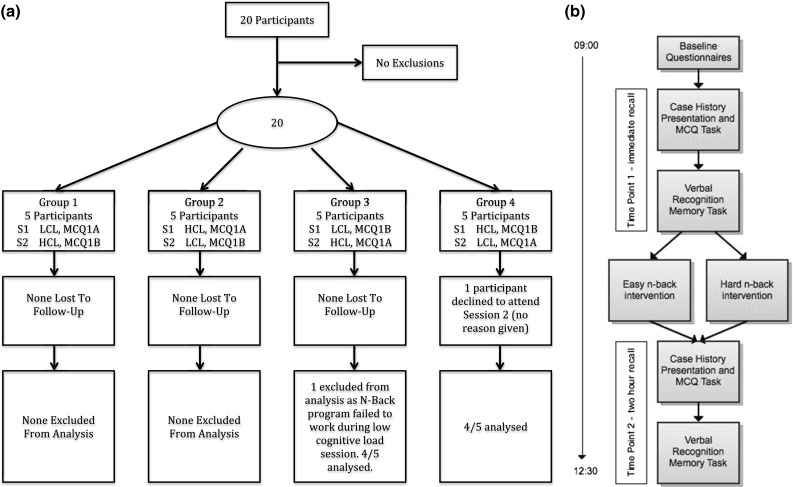
**a** Consort diagram. A crossover design study involving 20 participants. Participants were randomised to 1 of 4 Groups at session 1 (S1) that had been allocated to high (HCL) or low (LCL) cognitive loads. MCQ’s at pre and post cognitive loading were reversed at session 2 (S2). At the end of the study, 1 candidate was excluded from Group 3 and Group 4. **b** Study protocol. Participants completed baseline questionnaires prior to reading 5 Case Histories for a 20 min time period. Free and Cued recalls were observed consecutively for clinical and non-clinical information at ‘Assessment 1’. Easy or hard 90 min cognitive loading was performed according to allocation. Once completed, participants repeated a Free and Cued Recall assessment task in an identical order to previous at ‘Assessment 2’. The timings indicated on the left of the diagram represent the average time for participants to complete one session

20 medical students familiar with case history presentation skills were recruited to a two-day randomised-crossover study between February and August 2012 (Fig. 1a, b). Randomisation was by a blinded sealed brown envelope allocation to one of four groups based upon cognitive load and questionnaire assessment (Fig. 1a).

The study was performed in an empty quiet, room. All participants were required to be healthy individuals taking no regular medications, and free from stimulants (caffeine) from midnight prior to the study.

Prior to commencing the study, participants completed a National Adult Reading Test (NART) [9], baseline demographics and health questionnaire. A Confidence questionnaire, Stanford Sleepiness Scale [10] investigating subjective fatigue and Visual Analogue Score (VAS) [11] assessing sixteen dimensions of opposing conditions (e.g. alert-drowsy), were completed before each session.

Participants completed baseline questionnaires at 0930 h followed by a 20-min memorisation task. Candidates were allocated 4-min per case history (in a specific order). Time Point-1 baseline assessments of Free recall of clinical information, Multiple Choice Questions and the non-clinical Verbal Recognition Memory task (VRM-CANTAB-Cambridge Cognition Limited, Cambridge, United Kingdom) [12]were then performed. The cognitive loading task (N-Back)—level dependent on randomisation, immediately followed, with the VAS and NASA-TLX questionnaires subsequently performed pre and post Time Point-2 assessments.

## Case histories

Ten case histories with similar diagnoses were generated (Appendix 1). Participants were allocated five cases to memorise, different for each study session. Case histories were designed using a structured approach, for example –pain would be described in a standard manner—initial site of pain, current site, rapidity of onset, type of pain, exacerbating and relieving factors, prior to describing other symptoms. This format allowed point allocation for each statement, with no distractors (information not assessed).

## Multiple choice questionnaire (MCQ)

Ten sets of two 20-question, multiple choice questionnaires were developed corresponding to the Case History Information sheets (Appendix 1). Answers could consist of wrong information(confabulated/information from a different history), correct information or the answer ‘other’- enabling questions to be asked that contained two wrong answers and ‘other’ being the correct answer (Appendix 2).

## Time point 1: immediate assessment

Participants were provided with five case histories with 4-min to memorise each case in a consecutive order. Each case history was removed at the end of this allocated time.

### Assessments

#### Clinical

Free (verbal) recall was assessed by recording and marking the presentation of all five case histories. Cued recall—information memory cues to aid recall, was assessed using MCQ’s. They were assessed in a different order to the memorisation task for each time point and session (identical for all participants).

#### Non-clinical information

Recall of non-clinical information was assessed using the VRM test. Candidates memorised a set of eighteen words presented on a monitor prior to their immediate free recall. Recognition memory cues comprised of thirty-six words individually displayed (18 correct/incorrect words to act as cues to recall). Two sets of the VRM task were used to increase the amount of information and thus difficulty of recall.

### Cognitive loading and fatigue-inducing interventions

The N-Back task [13] is a continuous performance task. A white square on a grey background randomly appears at one of eight positions on the computer screen every 2.5 s, that is visible for 0.5 s. The participant indicates when the current stimulus matches that from *n* steps back in the sequence.

Depending on randomisation the task was delivered at a high/low cognitive load setting. The low cognitive loading task involved the recall of the square 1 step back. Subjects performed this task for 15-min alternating with 15-min rest for 90-min. The high cognitive loading (Dual-2-Back) task involved the presentation of two independent sequences simultaneously (audio of letters & visual squares) matching each stimulus from 2 steps back. This task was performed in 15-min blocks continuously for 90-min.

## Time point 2: post intervention recall assessment

Identical methodology was followed (as that in Time Point 1) for the assessment of clinical and non-clinical free and cued recall information.

### Statistics

Statistical analysis was performed using Stata/SE 12 (StataCorp) [14]. Normality was assessed using the Shapiro–Wilk test. The Wilcoxon Sign Rank test was used to analyse data between the High and Low Cognitive load groups for subjective questionnaires, free and cued recall (for related data).

## Results

20 medical students were recruited. One withdrew after one session and one was excluded due to failure of the N-Back program. Remaining participants were matched on all measured baseline parameters. There was no significant difference in age or clinical year of study between groups who started with the low (LH) or high (HL) cognitive load task (Table 2 in Appendix 3). Attitude and confidence scores performed prior to low and high cognitive load tasks were similar (LH = 55.06 se2.55, HL = 54.67 se2.33, *p* = 0.65). The mean NART result was 116.19 (se0.60). One subject omitted the NART during the study and subsequently could not be contacted. As each candidate is their own control this did not impact on analysis of the results.

### Cognitive loading

**Fig. 2:**
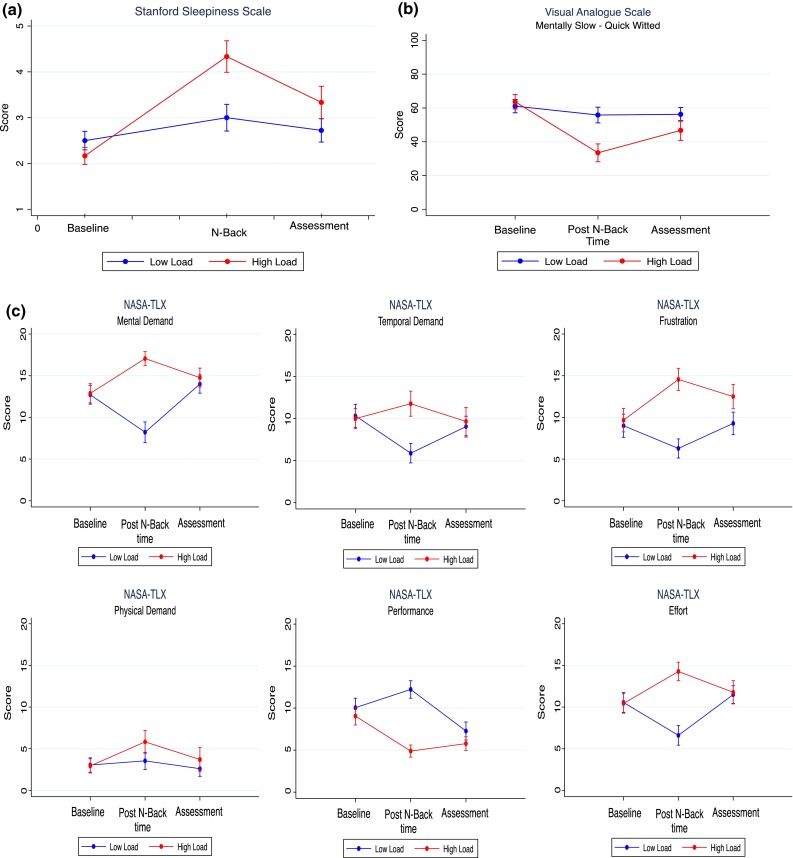
**a** Stanford sleepiness scale. The Figure displays subjective levels of sleepiness at baseline, intervention with the N-Back task (High or Low Load) and after Assessment., with* error bars* displaying standard error.** b** Visual analogue scale. Subjective rating of level of mentally slow (0) to Quick Witted (100). Error bars = standard error.** c** Graphic representation of NASA-TLX questionnaire. Error bars = standard Error

High cognitive loading (high cognitive load = HCL, low cognitive load = LCL induced subjective fatigue (Table 1). This was demonstrated through a significant decrement in wakefulness (*p* < 0.001) (Stanford Scale Fig. 2a), participants feeling mentally slower (Fig. 2b) (*p* < 0.001) and less attentive (*p* < 0.01) on the VAS, and increased mental demand (*p* < 0.01), effort (*p* < 0.01), performance (*p* < 0.001) and frustration [*p* < 0.001 (Fig. 2c)] on the NASA-TLX scale.

**Table 1:** Results for VAS and NASA-TLX for LCL and HCL at time points “post cognitive load” and “2 h free recall”

VAS (a.u.)	Post cognitive load			2 h free recall assessment		
	LCL (se)	HCL (se)	*p*	LCL (se)	HCL (se)	*p*
Mentally slow–quick witted	55.82 (4.52)	33.47 (5.12)	*p* < 0.001	56.23 (3.90)	46.72 (5.72)	*p* = 0.089
Attentive–dreamy	48.23 (5.23)	64.86 (6.09)	*p* < 0.010	42.04 (5.33)	51.00 (6.03)	*p* < 0.050
Incompetent–proficient	58.70 (4.51)	37.75 (4.84)	*p* < 0.001	56.09 (4.22)	43.78 (5.13)	*p* < 0.050
Withdrawn–gregarious	52.54 (4.24)	33.33 (4.15)	*p* < 0.010	56.63 (4.56)	40.76 (4.92)	*p* < 0.010
Alert–drowsy	44.11 (4.82)	67.00 (5.79)	*p* < 0.010	36.55 (5.14)	46.32 (6.31)	*p* < 0.010
Happy–sad	33.80 (4.89)	55.09 (6.11)	*p* < 0.001	38.62 (5.48)	46.45 (6.00)	*p* = 0.107
NASA-TLX (a.u.)						
Mental demand	8.22 (1.20)	17.06 (0.82)	*p* < 0.001	14.00 (1.07)	14.78 (1.11)	*p* = 0.282
Temporal demand	5.89 (1.13)	11.78 (1.45)	*p* < 0.010	9.06 (1.21)	9.67 (1.62)	*p* = 0.392
Frustration	6.28 (1.11)	14.56 (1.30)	*p* < 0.001	9.28 (1.32)	12.50 (1.42)	*p* < 0.050
Physical demand	3.56 (1.00)	5.83 (1.33)	*p* < 0.010	2.61 (0.89)	3.72 (1.37)	*p* = 0.289
Performance	12.22 (1.01)	4.89 (0.70)	*p* < 0.001	7.28 (1.05)	5.78 (0.79)	*p* = 0.238
Effort	6.61 (1.14)	14.28 (1.08)	*p* < 0.010	11.50 (1.03)	11.78 (1.36)	*p* = 0.570

### Free recall: clinical and non-clinical information

**Fig. 3:**
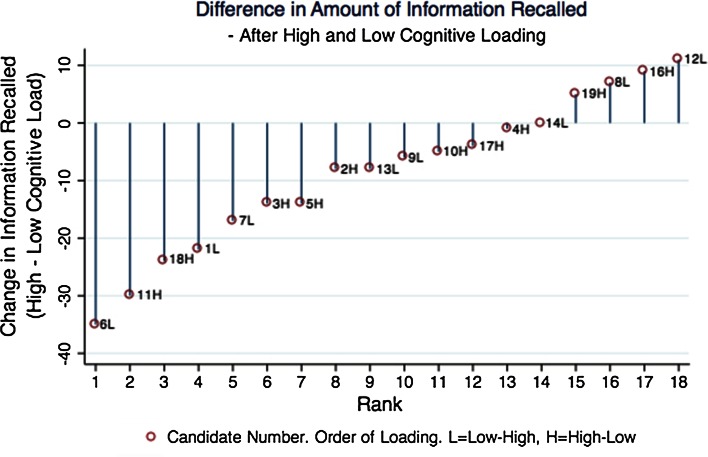
The pairplot displays the difference in information recalled per subject under high and low cognitive load. Value for each condition was the difference in amount of information recalled pre and post N-Back task

The change in the amount of information that is freely recalled is significantly reduced by increased cognitive load [mean decrease in information (a.u.) following LCL = 1.33 se2.81, following HCL = 10 se2.33, *p* < 0.05]. The pair plot depicted in Fig. 3 demonstrates a subgroup of individuals who have poor recollection of information when acutely fatigued. Participants identified in this group could deliberately incorporate the use of mental aids at work to aid recall of information if this was demonstrated to them. There was no significant difference in the change of freely recalled non-clinical information between sessions (mean change (a.u.) post LCL = 4.44 se0.94, HCL = 6.11 se1.01, *p* = 0.39).

### MCQ recall: clinical and non-clinical information

There was no significant change in amount of information recalled under low and high cognitive load conditions for the MCQ task for recall of clinical information [mean decrease in cued recall (a.u.) LCL = 2.56 se1.76, HCL = 2.44 se1.61, *p* = 1]. The mean amount of correct recall pre-cognitive loading was 61.28 % (se1.98) for the low load session, and 60.17 % (se2.73) for the high load session. Following the N-Back task recall was 58.72 % (LCL se2.69) and 57.72 % (HCL se2.79) of the correct information. There was no significant change in amount of information recalled between sessions for low and high cognitive load conditions for the recall of non-clinical information [mean decrease in non-clinical information recall (a.u): LCL = 2.78 se0.87, HCL = 3.89 se1.05, *p* = 0.08].

## Discussion

This study found deterioration in free recall of information with increased fatigue-inducing cognitive load, but displayed relative sparing of recognition memory and cue-induced recall in multiple choice questions. Confidence in the accuracy of recalled information was maintained with the latter assessment (Appendix 4).

Evidence from cognitive psychology demonstrates that memory cues aid in information retrieval [15, 16]. The current study is consistent with the literature indicating that recognition memory and cued recall are superior to the free recall of information [17]. This study is the conscious application of cognitive psychology to improve dissemination of medical information.

However, one should still view the results in context. This was a study of medical students fatigued by a non-medical cognitive task for 90-min and assessed on their recall of five case histories. It has positive findings that need further investigation. It is likely that memory retrieval of clinical information is less robust in medical students who have less clinical understanding (Levels of Processing theory) [18]. The study would need further validation within clinicians to confirm current findings. Equally different experience levels may reveal different findings, thus it should be targeted at the appropriate level of doctors (i.e. if designing a handover proforma—the level of doctors involved in producing the handover document). One should also consider that a non-clinical cognitive fatiguing task may not evoke similar levels of cognitive fatigue that parallel a clinical setting. Whether the fatigue is task specific is also unknown. A translational study comparing the effects of the N-back to an on-call session would be of benefit.

Recall of information relies on the strength of the memory pathway that was formed when encoding the memory. The weaker the link, the more difficult the retrieval of the required information. Cued Recall occurs when the brain receives a clue to accessing the desired answer. To be an effective stimulus, the cue must relate to the way the subject interpreted the information at the time of consolidation. For example, if interpretation of the word ‘foot’ were anatomical, one would not immediately associate the word to the cue ‘distance’. If the interpretation of ‘foot’ was that of a measurement, then it would be a helpful cue. Recognition memory has increased specificity where the response to a presented item of information would be a yes or no response.

An individuals interpretation of information at the time of encoding can significantly effect recall [19]. Thus the experience of the medical practitioner at the consultation will effect what is recalled. Hence, a proforma using memory cues is more likely to aid the less experienced clinician. In our opinion, it is the less experienced doctor who is involved in dissemination of information at handover, and a proforma could be designed to produce information that an experienced clinician would want.

Checklists use recognition memory and cued recall to stimulate actions or information retrieval. They can prevent deliberate omission of information or aspects of a procedure—‘cutting corners’ due to over familiarity or fatigue. The safety conscious airline industry uses checklists for this purpose. A benefit of this type of fatigue-resistant process is that less experienced staff can produce an identical output of critical information by following the checklist format. This cognitive process has been applied to the WHO pre-operative checklist [20].

**Fig. 4:**
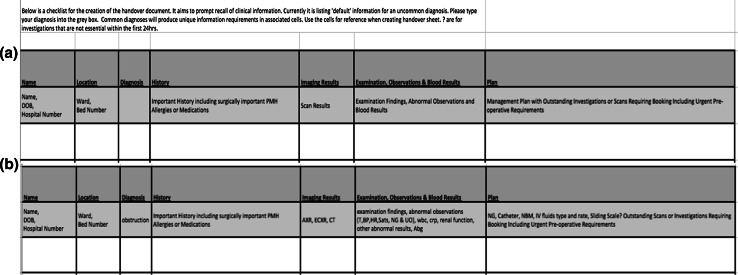
Implementation of cue-based recall in a current unpublished study. Participants are provided with memory cues based on aspects of information desired at handover dependent on input of diagnosis.** a** Information displayed prior to entering diagnosis.** b** The display changes on the input of the diagnosis, with information specific to condition displayed. The participant does not have to input all of the information displayed, the principle is to create a cue so that all of the information is considered

Cues can trigger information recall of the required and related information. Within medicine, we sub-consciously use serial recall (the recall of related information in a memorised order) as a cue to presenting patient information. If a surgeon was to recall a patient with small bowel obstruction, the free recall of the diagnosis should trigger serial recall of classical symptoms, e.g. feculent vomiting with burping and absolute constipation, that would act as cues for specific patient information. However, as shown in this study, this process is susceptible to cognitive fatigue. Medical handover has the potential to generate evidence-based written cues to aid recall of information beyond the current written format. A handover system could be developed to use cues and recognition memory to improve dissemination of the required information by less experienced staff (example of a cue-based handover system Fig. 4).

Free recall of clinical information is significantly reduced by a cognitively demanding 90-min task. It is conceivable that errors in patient handover may be made in a short time between clerking a patient and presenting the history, even in the non-sleep-deprived state. Extrapolating upon these findings, it is feasible that any cognitively demanding clinic or operative procedure could result in impaired recall.

The quantity of information desired at handover is a defining factor to the accuracy and safety of the process. The use of high volume data over a short time period could create information displacement from memory (as described by the Model of Working Memory) [21], a problem likely to be faced by on-coming teams during handover. The results raise interesting questions concerning how the profession should approach the dissemination of information. If the volume of information is high, then it is likely that some information will be lost, however, if a minimalistic approach is taken, crucial information may be omitted. It is clearly a difficult balance. Handover requires an evidence-based, fatigue-resistant process that achieves efficient, dissemination of the smallest amount of accurate patient information for a practical safe transition of care.

Checklists specifically designed to resist fatigue-related errors could be beneficial in a modern healthcare system. The implementation of the WHO checklist [20] is a move in this direction. Cognitive fatigue is not restricted to information recall but broadly influences a doctor’s ability to provide quality care. Fatigue may influence any aspect of care, for example vigilance and polyp detection rates during colonoscopy [22]. What time limit or number of procedures should be performed in a session before time-on-task effects significantly impair performance? Future work should address these important questions.

## Conclusion

This study has identified a sub-group of individuals that are significantly affected by acute mental fatigue. When fatigued, free recall of clinical information is significantly impaired. The relative preservation of recognition and cued recall allows for the design of checklists that can trigger recall of essential areas of information during handover. With work hour restrictions limiting but not eradicating work-based fatigue, targeted interventions need to be developed to limit fatigue-related error. Patient handover has specifically been identified as an area of concern [4]. Implementation of a generalised checklist that uses memory cues could create a baseline standard from which improvement in patient care can be developed.

## Figures and Tables

**Fig. 5:**
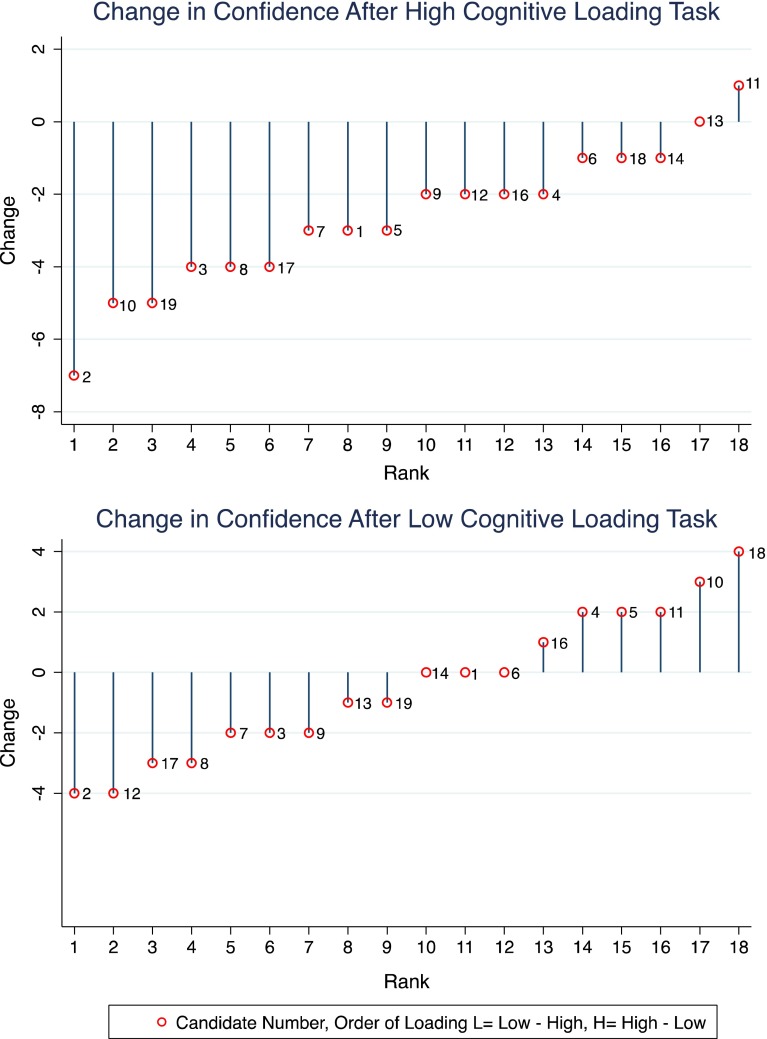
‘Change in Confidence’ after the N-Back task (top = High Load, Bottom = Low Load). A subgroup of six subjects display a possible susceptibility to reduced confidence when under cognitive demand. Six of the lowest nine ranked subjects display reduced confidence after high or low cognitive loading

## Appendix 3

See Table 2.

**Table 2:** Mean baseline demographics with standard deviation (se) shown in brackets

Randomisation group (cognitive loading)	Age (years)	Clinical year (year)	Attitude and confidence score (a.u.)
Low–high	22.68 (0.255)	4.111 (0.261)	55.056 (2.550)
High–low	23.326 (0.572)	4.111 (0.261)	54.667 (2.325)
	*p* = 0.260	*p* = 1	*p* = 0.646

## Appendix 4

### Confidence

There was no significant difference in confidence when presenting case histories prior to the cognitive loading task (mean low cognitive load 12.22 se0.65, mean high cognitive load 12.67 se0.63, *p* = 0.63). Following the N-back task, there was a significant difference between sessions for the change in the confidence in the accuracy of the free recalled clinical information. Specifically, subjects were observed to be less confident following the high load condition i.e. when mentally fatigued [mean change—low load 0.44 se0.55, high load = -2.67 se0.45, *p* < 0.05]. The confidence of a subgroup of participants is particularly effected by any cognitive loading. This does not necessarily reflect a decrement in knowledge, when compared to Fig. 3.

There was no significant difference between sessions for the change in confidence for cued recall (mean decrease in confidence score—low load 16.11 se11.53, high load 14.06 se14.40, *p* = 1).

### Change in confidence after high and low cognitive loading

See Figure 5.

## References

[1] World Health Organisation. Patient safety. Report for methods and measures working group of WHO patient safety. [World Health Organisation website] April, 2009. http://www.who.int/patientsafety/research/methods_measures/human_factors/human_factors_review.pdf. Accessed 31 July 2013

[2] Nasca TJ, Day SH, Amis ES, ACGME Duty Hour Task Force (2010). The new recommendations on duty hours from the ACGME Task Force. N Engl J Med.

[3] Union E (2003) Directive 2003/88/EC of the European Parliament and of the Council of 4 November 2003. Off J Eur Union 9–19

[4] The Patient Saftey Advisory Group (2011). Health care worker fatigue and patient saftey. Jt Comm: Sentin Event Alert.

[5] The Joint Commission. The Joint Commission: action urged to fight health care worker fatigue. *pwrnewmedia.com* 1–3 (2011). http://www.pwrnewmedia.com/2011/joint_commission/sea_fatigue/

[6] Machi MS (2012). The relationship between shift work, sleep, and cognition in career emergency physicians. Acad Emerg Med.

[7] Orton DI, Gruzelier JH (1989). Adverse changes in mood and cognitive performance of house officers after night duty. BMJ.

[8] Tanaka M, Mizuno K, Tajima S, Sasabe T, Wantanabe Y (2009) Central nervous system fatigue alters autonomic nerve activity. Life Sci 235–239. http://www.sciencedirect.com/science/article/pii/S0024320508004955 10.1016/j.lfs.2008.12.004 19100749

[9] Nelson HE, O’Connell A (1978). Dementia: the estimation of remorbid intelligence levels using the New Adult Reading Test. Cortex.

[10] Hoddes E, Zarcone V, Smythe H, Philips R, Dement WC (1973). Quantification of sleepiness: a new approach. Psychophysiology.

[11] Bond A, Lader M (1974). The use of analogue scales in rating subjective feelings. Br J Med Psychol.

[12] Sahakian BJ, Owen AM (1992). Computerized assessment in neuropsychiatry using CANTAB: discussion paper. J R Soc Med.

[13] Kirchner WKW (1958). Age differences in short-term retention of rapidly changing information. J Exp Psychol.

[14] Stata Statistical Software. Version 12. College Station, TX: StataCorp LP, 2011

[15] Mantyla T (1986). Optimising cue effectiveness: recall of 500 and 600 incidentally learned words. J Exp Psychol.

[16] Hunt RR, Smith RE (1996). Accessing the particular from the general: the power of distinctiveness in the context of organization. Mem Cognit.

[17] Tulving E, Brown J (1976). Ecphoric processes in recall and recognition. Recall and Recognition.

[18] Craik FI, Lockhart RS (1972). Levels of processing: a framework for memory research. J Verbal Learn Verbal Behav.

[19] Winter L, Uleman JS (1984). When are social judgments made? Evidence for the spontaneousness of trait inferences. J Pers Soc Psychol.

[20] Haynes AB (2009). A surgical safety checklist to reduce morbidity and mortality in a global population. N Engl J Med.

[21] Baddeley A, Hitch G (1974). Working memory. The psychology of learning and motivation.

[22] Harewood GC, Chrysostomou K, Himy N, Leong WL (2008). Impact of operator fatigue on endoscopy performance: implications for procedure scheduling. Dig Dis Sci.

